# IN-HOME ASSISTIVE TECHNOLOGY PROTECTS DEMENTIA CAREGIVERS FROM WORSENING SLEEP EFFICIENCY

**DOI:** 10.1093/geroni/igae098.4271

**Published:** 2024-12-31

**Authors:** Julian Scheffer, Darius Levan, Jenna Wells, Dolores Gallagher-Thompson, Kevin Grimm, Kuan-Hua Chen, Gene Wang, Robert Levenson

**Affiliations:** University of Western Ontario, London, Ontario, Canada; University of California, Berkeley, Berkeley, California, United States; Cornell University, Ithaca, New York, United States; Stanford University, Stanford, California, United States; Arizona State University, Tempe, Arizona, United States; University of Nebraska Medical Center, Elkhorn, Nebraska, United States; Care Daily Company, Kirkland, Washington, United States; University of California, Berkeley, San Francisco, California, United States

## Abstract

Caregivers for people with dementia or mild cognitive impairment often report sleep problems due to heightened vigilance concerning worrisome behaviors (e.g., falls, wandering) by their care recipients (CRs). Interventions are needed to help alleviate these issues and the associated sleep troubles for caregivers. One promising approach is to utilize scalable and affordable in-home technologies that monitor CR behaviors round-the-clock and alert caregivers to potentially dangerous situations. We conducted two randomized controlled trials with independent samples to determine whether People Power Caregiver (PPCg), a newly developed in-home monitoring and alerting system, benefitted caregivers’ sleep over a six-month period. Combining the two studies, a total of 162 primary caregivers of the CRs were randomly assigned to either an active condition (PPCg system fully activated) or a control condition (Study 1: water leak detection only; Study 2: waitlist control procedure). Caregivers self-reported their sleep quality using the Pittsburgh Sleep Quality Index at baseline, three-months, and six-months. Using latent growth modeling, caregivers in the control conditions reported significantly worsening sleep efficiency compared to caregivers in the active condition (Active: B = -0.30, SE(B) = 0.14; Control: B = 0.74, SE(B) = 0.12; Condition Effect on Linear Slope: p =.038). We observed a similar pattern with PPCg benefitting caregivers’ sleep duration; however, this result was not statistically significant (Condition Effect on Linear Slope: p =.105). These results suggest that in-home technologies such as PPCg may offer an effective way to help caregivers achieve healthy sleep as they contend with the demands of caregiving.

